# Steady-state burning plasma: a new stage in the development of magnetic confinement fusion energy

**DOI:** 10.1093/nsr/nwad217

**Published:** 2023-08-14

**Authors:** Baonian Wan, Guosheng Xu

**Affiliations:** Institute of Plasma Physics, Chinese Academy of Sciences, China; Institute of Plasma Physics, Chinese Academy of Sciences, China

**Keywords:** Steady-state burning plasma, magnetic confinement fusion, tokamak, high-temperature superconductor, strong magnetic field, DT fusion

## Abstract

Over the past 20 years, advances in tokamak physics and technology have prepared the field of magnetic confinement fusion research for the next step toward a steady-state burning plasma.

In the last century, the fusion triple product $nT{\tau }_E$ (density, ion temperature and energy confinement time) obtained on tokamak devices have increased faster than the ‘Moore's law’ in the field of chips. However, after the JET, TFTR and JT-60 U three large devices, the international joint construction of the International Thermonuclear Experimental Reactor (ITER) began. No larger device has been built yet, so $nT{\tau }_E$ stagnates, as shown in Fig. 1(a). Deuterium and Tritium (DT) burning plasma experiments have so far only been conducted on JET ($Q \sim 0.3$ for 5 s in 2021) and TFTR tokamaks, and ITER is not scheduled to begin DT burning plasma experiments until 2035. Although there is no larger device, two fully superconducting tokamaks, EAST and KSTAR, were built with the intention of solving the physical and technical challenges associated with tokamak steady-state operation. Advanced physics and technology on these tokamaks have made great progress in the last 20 years, and their combination has shown promise for breakthroughs, leading to increased investment in fusion energy development from governments and capital markets in recent years, with many countries making plans to build Fusion Pilot Plants (FPPs) [1].

**Figure 1.**
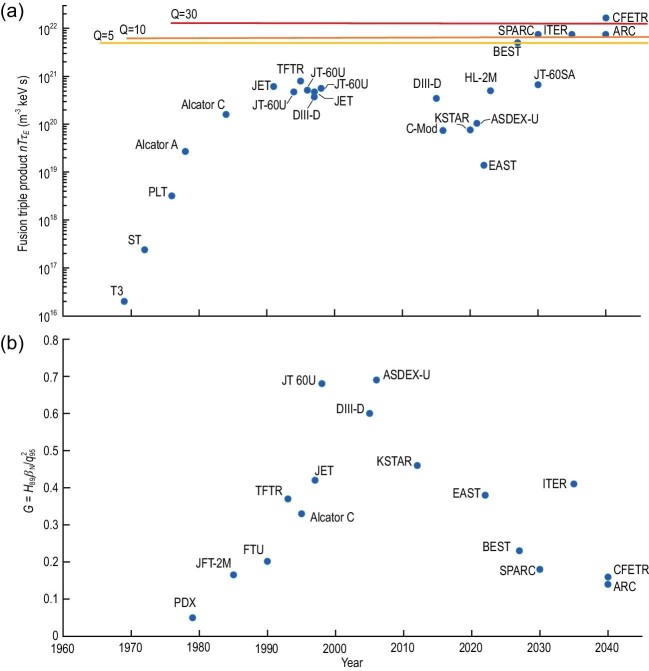
(a) Improvement of the fusion triple product (relative units) with time as tokamaks of increasing size, field and plasma current are constructed and brought into operation. (b) The normalized fusion gain factors *G* achieved on current experimental devices have exceeded the values required for future DT fusion devices, such as ITER, CFETR, BEST, SPARC and ARC.

Generation of burning plasma requires high fusion triple product $nT{\tau }_E$, i.e. high pressure $p\ = \ nT$ and high energy confinement. Plasma pressures close to those expected on ITER have been achieved by the high-field compact tokamak Alcator C-Mod. And, normalized fusion gain factors $G = {H}_{89}{\beta }_N/q_{95}^2 \ge 0.4$ have been achieved on current experimental devices, exceeding the values required for ITER’s baseline operating scenario, as shown in Fig. 1(b), which means that the physical conditions required for tokamak fusion are largely in place. Here, ${H}_{89}$ is the energy confinement enhancement factor relative to the international tokamak energy confinement scaling in 1989, ${\beta }_N = \beta aB/{I}_p$ is the normalized pressure and ${q}_{95}$ is the safety factor at the 95% poloidal flux surface with $\beta $ being the plasma pressure ratio, *a* being the plasma minor radius, *B* being the magnetic field strength and ${I}_p$ being the plasma current. Extrapolation from these existing experimental results and physics-based models have given confidence that burning plasmas will be accessible in the next-step devices. Because $nT{\tau }_E \propto G{B}^3{a}^3$ [2], it means that achieving a high fusion triple product requires a coupling of physics and engineering, i.e. it requires simultaneous optimization of ${\beta }_N$, ${q}_{95}$, energy confinement performance, magnetic field strength and device size.

In terms of technology, the rapid development of magnet technology based on the second-generation High-Temperature Superconductor (HTS) tapes is expected to double the magnetic field strength of tokamak [3]. Under similar physical conditions, i.e. for similar values of *G*, the plasma major radius *R* is expected to be reduced to half, and the cost of the device is expected to be reduced by nearly one order of magnitude compared with ITER. Recently, CFS, an American company, has designed a strong-field tokamak SPARC for short-pulse operation based on HTS, which is only 1/80 the volume size of ITER, but aims to achieve the same fusion gain $Q\ = \ 10$ as ITER [4]. Furthermore, an innovative bucking design that integrates the Toroidal Field (TF) coils and the center solenoid effectively reduces the radial size and stress of the TF coils on the high-field side, making the device more compact. Next, CFS plans to build ARC, a compact FPP, which was originally proposed as an advanced tokamak steady-state device to generate net electricity [3]. It adopts an innovative design of demountable TF coils to facilitate the replacement of internal components and overcome the problem of degraded performance of neutron-irradiated materials. Based on the relevant concepts, the US and UK governments are actively advancing the design of FPPs [1,5]. In addition, iron-based superconductors are also developing rapidly, and their mechanical properties are fundamentally better than the second-generation ceramic nature HTS materials, the cost is potentially low, but the technology has yet to break through.

The physical effects of strong magnetic fields may change the way tokamaks operate and facilitate access to the burning plasma regime [3], such as: (1) Allowing for higher plasma densities ${n}_{GW} \propto B/R{q}_{95}$, and thus higher plasma pressures. (2) Allowing tokamaks to operate at a higher ${q}_{95}$, thus reducing the risk of plasma major disruption and the amplitude of the Edge Localized Modes (ELMs, imposing transient heat load on material surfaces), allowing for safer operation. (3) Making it easier to obtain a high bootstrap current fraction ${f}_{BS} \propto {\beta }_N{q}_{95}$, which reduces recirculating power. Here, bootstrap current is a plasma self-generated current driven by density and temperature gradients. (4) Less impurity fraction ${f}_z \propto {B}^{ - 0.97}$ being required to achieve divertor detachment, preventing high heat and particle fluxes toward divertor plates. (5) With a smaller poloidal ion gyroradius ${\rho }_{\theta i} \propto {q}_{95}/B$, fast ion losses, neoclassical transport and turbulent transport tend to be smaller. (6) Improvement in current drive efficiency. (7) The center solenoid providing more volt seconds. Despite these benefits of a strong magnetic field, there are some new physical difficulties: (1) The heat flow ${q}_ \bot \propto B/R{q}_{95}$ toward the divertors is even more challenging. (2) The required power for transition from low-confinement mode to high-confinement mode ${P}_{LH} \propto {n}^{0.7}{B}^{0.8}$ is higher. (3) The synchrotron radiation loss ${P}_{syn} \propto {B}^{2.6}$ is larger. On the technical side, high stress in magnets under strong magnetic fields challenges superconducting and structural materials. Also, there are few mature heating and current driving methods suitable for a strong magnetic field and high density, e.g. high-frequency high-power electron gyrotron suitable for >7T strong magnetic field is currently unavailable. The lower hybrid wave and neutral beam injection have low current driving efficiency at high density, and their radial deposition positions are close to the plasma edge. Efficient current drive by ion cyclotron wave has yet to be verified and there are problems with strong interaction between plasma and antennas.

The greatest progress in physics in the last 20 years has been the development of several advanced steady-state operating scenarios with high ${\beta }_N$ on tokamaks [2], and long-pulse fully non-inductive H-mode operation with high ${f}_{BS}$ has been demonstrated [6]. The improved control of current, pressure profiles and MHD stabilities makes it possible to operate in a stable manner near the ${\beta }_N$ no-wall limit, which refers to the MHD stability limit in the absence of the stabilization effect of a surrounding conducting wall. Other important developments include: (1) compatibility of high-performance core plasma with divertor detachment [7]. (2) High-confinement operation beyond the Greenwald density limit being achieved with deep fueling. (3) Super-H mode with high pedestal density and pressure being discovered. (4) Development of ELM solutions such as the small-ELM regime and the resonant magnetic perturbations. (5) Development of an advanced divertor. (6) Development of integrated modeling enhances predictability. In addition, artificial intelligence has also penetrated into the field of magnetic confinement fusion research in recent years. It has been successfully applied in major disruption prediction, plasma control [8,9], mechanism mining, large-scale numerical simulation, and material development, etc.

Solving the above-mentioned coupling and integration of engineering and physics, and the self-consistency of different physical aspects is still not a simple task. It still needs a lot of R&D to find the solution for compatible integration among the different aspects discussed above. Burning plasma brings new opportunities and challenges to the field of magnetic confinement fusion [10]. The nonlinear interactions of waves, instabilities and turbulences with energetic particles will lead to a wealth of new physics. The nonlinear self-organizing system formed through these interactions will produce new control methods of parameter profile through alpha channeling, such as heating, rotation drive, energetic particle losses and Internal Transport Barrier (ITB) formation, thus generating new steady-state operating scenarios. The enhancement effect of energetic particles and their instabilities on energy confinement and fusion reaction rate may facilitate fusion energy production. For example, energetic particles have been observed to facilitate turbulence suppression and ITB formation [11]. Energetic particles can not only suppress instabilities such as sawtooth, but also can use energetic particle-driven instabilities, such as fishbone, to induce redistribution of plasma pressure and current to form a self-organized stable structure, resulting in high-confinement performance. This self-organized structure is less dependent on external control and thus is an ideal stable operating scenario for future fusion reactors. Moreover, there are many technological innovations to be developed, such as the high-field-side ion cyclotron wave and lower hybrid wave injection and other new heating schemes suitable for strong-field conditions.

In short, the advances in physics and technology on tokamak in the past 20 years have prepared the fusion field for the next step toward achieving steady-state burning plasma. In particular, the emergence of new technologies such as a strong-field magnet has made magnetic confinement fusion a promising breakthrough. Magnetic confinement fusion research is about to enter a new stage of DT fusion, which will face new opportunities and challenges.
